# [Retracted] Oxymatrine-mediated maturation of dendritic cells leads to activation of FOXP3^+^/CD4^+^ Treg cells and reversal of cisplatin-resistance in lung cancer cells

**DOI:** 10.3892/mmr.2025.13725

**Published:** 2025-10-27

**Authors:** Hui Liu, Manman Zou, Pei Li, Haifeng Wang, Xijun Lin, Jin Ye

Mol Med Rep 19: 4081–4090, 2019; DOI: 10.3892/mmr.2019.10064

Following the publication of the above paper, it was drawn to the Editor's attention by a concerned reader that the flow cytometric data shown in Fig. 2A on p. 4085 were strikingly similar to data that had either already been published previously in articles that were written by different authors at different research institutes, or were featured in articles that were submitted for publication to different journals at around the same time. Owing to the fact that the contentious data in the above article had already been published prior to its submission to *Molecular Medicine Reports*, the Editor has decided that this paper should be retracted from the Journal. After contacting with the authors, they accepted the decision to retract the paper. The Editor apologizes to the readership for any inconvenience caused.

